# Increasing densities of an invasive polychaete enhance bioturbation with variable effects on solute fluxes

**DOI:** 10.1038/s41598-018-25989-2

**Published:** 2018-05-16

**Authors:** L. Kauppi, G. Bernard, R. Bastrop, A. Norkko, J. Norkko

**Affiliations:** 1 University of Helsinki, Tvärminne Zoological Station, J.A. Palménin tie 260, FI-10900 Hanko, Finland; 2 Department of Environmental Sciences, P.O. Box 65 (Viikinkaari 1), 00014 University of Helsinki, Helsinki, Finland; 3 CNRS, EPOC, UMR 5805, F33400 Talence, France; 4 University of Rostock, Institute of Biological Sciences, Albert-Einstein-Str. 3, D-18059 Rostock, Germany; 5 Baltic Sea Centre, Stockholm University, Stockholm, Sweden

## Abstract

Bioturbation is a key process affecting nutrient cycling in soft sediments. The invasive polychaete genus *Marenzelleria* spp. has established successfully throughout the Baltic Sea increasing species and functional diversity with possible density-dependent effects on bioturbation and associated solute fluxes. We tested the effects of increasing density of *M. arctia, M. viridis* and *M. neglecta* on bioturbation and solute fluxes in a laboratory experiment. Benthic communities in intact sediment cores were manipulated by adding increasing numbers of *Marenzelleria* spp. The results showed that *Marenzelleria* spp. in general enhanced all bioturbation metrics, but the effects on solute fluxes varied depending on the solute, on the density and species identity of *Marenzelleria*, and on the species and functional composition of the surrounding community. *M. viridis* and *M. neglecta* were more important in predicting variation in phosphate and silicate fluxes, whereas *M. arctia* had a larger effect on nitrogen cycling. The complex direct and indirect pathways indicate the importance of considering the whole community and not just species in isolation in the experimental studies. Including these interactions provides a way forward regarding our understanding of the complex ecosystem effects of invasive species.

## Introduction

Benthic invertebrates have a pivotal role in modifying biogeochemical properties and nutrient transformation processes at the sediment–water -interface^1–3^. Nutrient remineralization is a key ecosystem process providing the basis for primary production in pelagic and benthic food webs^4–6^. Remineralization processes are affected by a number of factors including temperature, organic matter quantity and quality, and the structure of benthic infauna (both macro- and micro-organisms), modified by seasonality in the system^7–10^. Macrofauna enhances remineralization processes through their bioturbation, which enhances the activity of the microbial communities in the sediment ultimately responsible for organic matter remineralization^2,9,11^, through oxygenating the sediment which enhances aerobic mineralization^12,13^, and by enhancing diffusion^1^. Bioturbation activities by benthic macrofauna include *all transport processes carried out by animals that directly or indirectly affect sediment matrices. These processes include both particle reworking and burrow ventilation*^14^. Differences in species richness and the species’ functional traits, as well as interactions among these determines their contribution to ecosystem processes, such as nutrient cycling/remineralization^15,16^ and may be even more important for ecosystem functioning than species richness alone^7,17–20^.

Motivated originally by the effect of species loss on ecosystems, biodiversity-ecosystem functioning (BEF) studies have concluded that increasing biodiversity, and especially functional diversity, enhances ecosystem functioning in experimental conditions^21,22^. Species invasions are feared for their potential negative consequences on biodiversity, even though this fear is often not evidence-based^23^. On the contrary, species invasions may even increase the species and functional diversity in some areas^24,25^, but the importance of this potential increase for ecosystem functioning remains largely unknown^22^ with further uncertainty about extrapolating effects from short-term experiments to longer-term effects in the ecosystem. Experimental studies on BEF-relationships offer important mechanistic understanding about the ecosystem responses to biodiversity change^26^, but can also be criticized for the highly controlled conditions used to reduce the natural variability that might confound the results of the mechanistic processes, even if some studies suggest that results from small-scale experiments are generalizable on a larger scale of observational studies^27^.

Invasive species are capable of a rapid population expansion in both space and time, and they can be either native or non-native. Increasing densities of a single species, however, increases the expression of the functional traits associated with this species, thus possibly affecting the ecosystem functioning depending on the studied process^5,24^. Recent field studies quantifying benthic oxygen and nutrient fluxes in the multiply stressed, highly invaded Baltic Sea have provided evidence of enhanced nutrient cycling in the sediment following the establishment of an invasive polychaete genus *Marenzelleria* spp.^10,28,29^. In total three species of the genus have become established and spread into the entire Baltic Sea since the first observations in the southern Baltic in 1985^30–32^, *M. viridis* and *M. neglecta* of North American, and *M. arctia* of Arctic origin^31^. In the study area, *M. arctia* prefers deeper (>20 m), muddy bottoms, whereas *M. neglecta* and *M. viridis* prefer shallower (up to 20 m) bottoms and *M. viridis* especially sandier areas^33^ Especially in the deeper, muddy sediments *M. arctia* can be a dominant member of the benthic communities along with the clam *Macoma balthica*, while all three of them have increased species richness and added functionality in the naturally species-poor benthic communities of the Baltic Sea^25^. Species of the genus *Marenzelleria* are classified as facultative deposit feeders and suspension feeders^34^. All three species in the Baltic Sea are classified as biodiffusers and have low particle reworking rates^35^. Compared to the native fauna, *M. viridis* and *M. neglecta* burrow deeper (down to 25–35 cm depth) into the sediment constructing L- or J-shaped burrows, whereas *M. arctia* burrows down to 6–8 cm depth and constructs a network of burrows (ref.^35^, pers. obs). *M. viridis* and *M. neglecta* affect the solute transport in the sediment mainly through non-local, advective transport, whereas the solute transport mode of *M. arctia* is of a more diffusive character^35^. Partly therefore, *M. viridis* and *M. neglecta* are also suggested to affect solute fluxes and nutrient cycling more than *M. arctia*^35,36^. These conclusions are, however, based on results from highly controlled, single-species experiments and do not necessarily reflect the species’ effects in a real-life context with variable community composition possibly affecting the outcome of BEF-relationships.

Phosphorus and nitrogen are the most important macronutrients for primary production, and play a key role in eutrophication. In the long run, increased density of *Marenzelleria* spp. could lead to increased burial of phosphorus in the sediment and thus mitigate eutrophication; increased density of the polychaetes is hypothesized to increase oxygenation of the sediment through bioturbation, which increases the availability of iron-oxyhydroxides that capture phosphate ions^37,38^. The fate of phosphorus is especially interesting, since the decreased internal phosphorus recycling results in decreased blooms of harmful cyanobacteria, which are phosphorus limited and plague the Baltic Sea ecosystem^39^. Increased oxygenation of the sediment could also facilitate the return of other macrofauna in previously hypoxic areas. The dominance of the *Marenzelleria* species complex in many areas in the Baltic Sea, and their potential importance for a key ecosystem process, nutrient cycling, also call for a mechanistic understanding of the species’ impact in the organic matter remineralization processes.

The effect of macrofauna on solute fluxes depends e.g. on density, biomass and functional traits of individual species and their interactions in the community^17,40,41^. In this study, we tested the density-dependence of the effect of *Marenzelleria* spp. on nutrient cycling in a density-manipulation experiment following the rationale in the model of Norkko & Reed *et al*.^38^. Their results show a release of phosphorus at moderate densities of worms (1000 to 3000 ind m^−2^) and a retention with population densities of over 3000 ind m^−2^. Thus, in a simple, single-species model, the direction and magnitude of P flux is dependent of *Marenzelleria* spp. density. In order to have as close a resemblance to natural conditions as possible, we conducted a density-manipulation experiment using intact sediment cores collected from the field. Our aims were to investigate 1) how an increasing density of *Marenzelleria* spp. affects fluxes of nitrate+nitrite (NO_x_) ammonium (NH_4_^+^), phosphate (PO_4_^3−^) and silicate (Si^4+^), 2) what bioturbation metrics are affected by *Marenzelleria* spp. and other macrofauna and 3) which bioturbation metrics are associated with the different solute fluxes?

## Results

### Experimental conditions

The sediment at the core collection site was classified as sand (D_50_ = 0.46 mm) with a C/N ratio of 7.05 and C content of 0.007%. The organic matter content of the sediment at the sediment collection site varies between 2.6 and 11.2% (measured as loss on ignition) throughout the year^10^. The surface sediment in the cores was a 3–4 cm layer of very fine silt, underneath which there was a 1–2 cm layer of gravel followed again by soft mud. Temperature in the water during the experiment varied between 12.5 to 14.2 °C and salinity varied between 5.5 and 5.8. Oxygen concentration in the cores ranged from 7.44 mg l^−1^ to 8.62 mg l^−1^, corresponding to a saturation of 75 to 86% during the experimental period. During the dark incubations the temperature in the incubation tank remained at approximately 14 °C, and salinity was 5.7.

### *Marenzelleria* spp. and other macrofauna recovered after the experiment

The community in the control cores represents the background community composition with hydrobid snails and *Marenzelleria* spp. as the most abundant species (see Table 1 for core-wise mean, min and max) followed by *Macoma balthica, Cerastoderma glaucum* and *Hediste diversicolor*. The clam *M. balthica* had the largest biomass (measured as g wet weight) in the control cores, followed by *Marenzelleria* spp., *H. diversicolor, C. glaucum* and the hydrobid snails. Other species occurring in the experimental cores were Chironomidae, *Pygospio elegans, Manayunkia aestuarina, Potamopyrgus antipodarum, Cyanophthalma obscura*, Oligochaeta, and some *Mya arenaria*, which could have a very large biomass. Of the *Marenzelleria* spp. added to the density manipulated cores 12.5% to 100% were recovered after the experiment. The low survival (12.5%) in one of the cores was due to large specimens of *H. diversicolor* present and feeding on the *Marenzelleria* spp. Excluding this core the percentage of added worms surviving was between 67 and 100%. The realized densities and biomasses of the most common infauna are presented in Table 1.

**Table 1:** Core-wise nutrient fluxes used as response variables, and macrofauna densities and biomasses, and bioturbation parameters used as predictors in the DistLM-analysis.

Core	NO_x_	NH_4_^+^	PO_4_^3−^	Si^4+^	*M. arctia*	*M. neg+vir*	*Marenzelleria*	*C. glaucum*	*M. balthica*	*H. diversicolo*	Hydrobiidae	D_b_^N^	SR	MPD	BI
	mmol m^−2^ d^−1^				ind m^−2^		ww g m^−2^					cm^2^ yr^−1^	%	cm yr^−1^	ml d^−1^
Ca	−0.02	−0.15	−2.08	−0.08	0	1805	23.43	0.00	155.56	0.00	3.65	0.73	83.23	6.50	14.10
Cb	0.05	0.07	0.50	0.62	211	1053	10.27	3.27	0.14	0.00	7.55	0.00	76.83	5.50	32.74
Cc	0.10	0.38	3.37	0.85	0	1625	15.00	7.38	24.62	0.09	5.13	5.84	82.79	5.50	41.35
Cd	0.45	0.46	86.82	4.90	181	0	1.93	0.00	186.82	49.69	5.32	2.19	57.56	8.50	10.98
1a	−0.01	0.46	52.85	0.29	812	812	21.34	0.00	238.50	3.61	3.19	5.84	68.57	10.00	78.89
1b	0.03	0.25	18.64	2.98	0	1264	1.75	0.00	6.84	4.89	5.05	1.46	74.49	5.50	36.31
1c	0.07	0.39	−4.22	0.10	542	0	2.71	9.35	0.00	3.77	5.07	1.10	58.86	8.50	21.72
1d	−0.02	0.81	4.59	−0.86	650	975	28.03	27.20	0.00	28.23	5.34	3.29	77.01	6.50	97.63
2a	0.09	0.61	18.89	0.78	421	843	41.75	6.61	0.00	0.45	4.68	0.73	79.12	5.50	95.07
2b	−0.06	0.14	8.52	0.41	316	948	7.17	2.40	183.93	0.00	2.33	0.37	69.50	5.50	37.63
2c	0.05	0.25	−2.31	0.04	1011	1517	13.32	2.33	0.00	0.00	2.69	1.10	68.69	4.50	22.53
2d	0.01	0.85	14.60	−0.45	963	481	10.79	2.24	0.02	0.00	3.09	1.46	71.63	12.00	57.80
3a	0.00	0.44	−1.69	0.17	1341	1006	33.57	2.17	0.00	2.94	2.94	2.19	80.05	7.50	14.27
3b	0.02	0.27	−1.60	1.26	963	481	4.95	1.23	0.02	0.00	1.23	0.37	65.73	3.50	54.24
3c	−0.02	0.51	0.57	−0.26	722	1805	35.18	11.99	124.48	0.00	7.91	1.46	69.93	8.50	45.77
4a	0.08	0.97	16.56	0.32	2949	843	34.01	8.10	173.39	0.00	5.23	4.02	80.06	5.50	67.37
4b	0.04	0.89	1.74	1.54	4198	1399	25.72	3.05	209.76	0.00	2.33	2.19	86.64	7.50	90.18
4c	0.02	0.93	8.11	−0.07	2785	464	20.63	3.92	0.00	14.10	0.99	1.10	80.98	10.00	60.01
4d	−0.02	1.04	3.76	0.93	2437	4062	65.47	2.92	0.00	0.00	2.40	4.75	88.35	8.50	131.25
5b	−0.09	1.39	12.11	1.65	9749	0	43.86	4.39	14.88	0.00	4.04	1.46	91.34	4.50	140.17
5c	−0.01	−0.70	−8.27	−2.19	433	650	14.58	42.64	9.64	164.87	3.63	2.56	90.30	8.50	103.81
average	0.04	0.49	11.02	0.62	1461	1049	21.69	6.72	63.27	12.98	3.99	2.11	74.70	7.05	59.71
min	−0.09	−0.70	−8.27	−2.19	0	0	1.75	0.00	0.00	0.00	0.99	0.00	25.83	3.50	10.98
max	0.45	1.39	86.82	4.90	9749	4062	65.47	42.64	238.50	164.87	7.91	5.84	91.34	12.00	140.17

Note that the values for *Marenzelleria* include both the natural community and the worms added as part of the density treatments. Bioturbation parameters D_b_^N^ = biodiffusion coefficient, SR = % of surface reworked, MPD = maximum penetration depth, BI = bioirrigation.

### Solute fluxes

NO_x_ fluxes in the experimental cores ranged from −0.092 mmol m^−2^ d^−1^ (high-density core 5b) to 0.446 mmol m^−2^ d^−1^ (unmanipulated core Cd), ammonium fluxes ranged from −0.704 mmol m^−2^ d^−1^ (high-density core5c) to 1.388 (high-density treatment 5b), phosphate fluxes ranged from −0.00827 mmol m^−2^ d^−1^ (high-density core 5c) to 0.0866 mmol m^−2^ d^−1^ measured in one of the controls (Cd), and silicate fluxes ranged from 2.186 mmol m^−2^ d^−1^ (in 5c) to 4.897 mmol m^−2^ d^−1^ (in Cd). Core-wise solute fluxes are presented in Table 1.

### Bioturbation metrics

**Figure 1:**
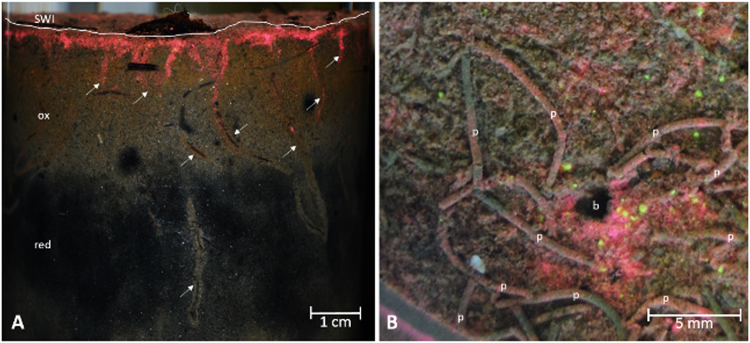
Photographs showing luminophore tracer fate consecutive to *Marenzelleria* spp. bioturbation and feeding activities at the end of the experiment. (**A**) Occurrence of *Marenzelleria* burrows (indicated by white arrows) burying tracers down in the sediment along glass-wall of core 5b. White line indicates the sediment-water interface (SWI). Sediment oxidized layer (ox) had a light brown color whereas reduced layer (red) was black. (**B**) Close-up photo from above core 2a showing the sediment surface with luminophore tracers (in pink and yellow), a *Marenzelleria* burrow opening (b) and fecal-pellet strings (p).

At the end of the experiment, burrow networks created by *Marenzelleria* spp. were seen along the core walls. Luminophore tracers were buried down into these burrows (Fig. 1A). Typical burrows created by *H. diversicolor* and siphonal gallery networks generated by *M. balthica* during deposit feeding and associated with tracer burial were also apparent. We could also observe fecal-pellet strings, typical of *Marenzelleria* spp., around the openings of the burrows at the sediment surface (Fig. 1B). These pellets were composed of both sediment and luminophore tracers, indicating that *Marenzelleria* spp. individuals had deposit-fed at the surface during the experiment.

The biodiffusion coefficient D_b_^N^ ranged from 0 (unmanipulated core Cb) to 5.84 cm^2^ d^−1^ (low-density manipulated core 1a). The percentage of surface reworked (SR) ranged from 57.6% (low-density manipulated core 1c) to 91.3% (high-density manipulated core 5b). The maximum penetration depth (MPD) ranged from 3.5 cm (core 3b) to 12.0 cm (core 2d). Bioirrigation (BI) had a minimum of 10.98 ml d^−1^ in the unmanipulated core Cd, and a maximum of 140.17 ml d^−1^ in the high-density treated core 5b. Core-wise bioturbation metrics are presented in Table 1.

### Pore water profiles and ΔNH_4_^+^, ΔNO_x_, ΔPO_4_^3−^ and ΔSi^4+^ during dark incubation

**Figure 2:**
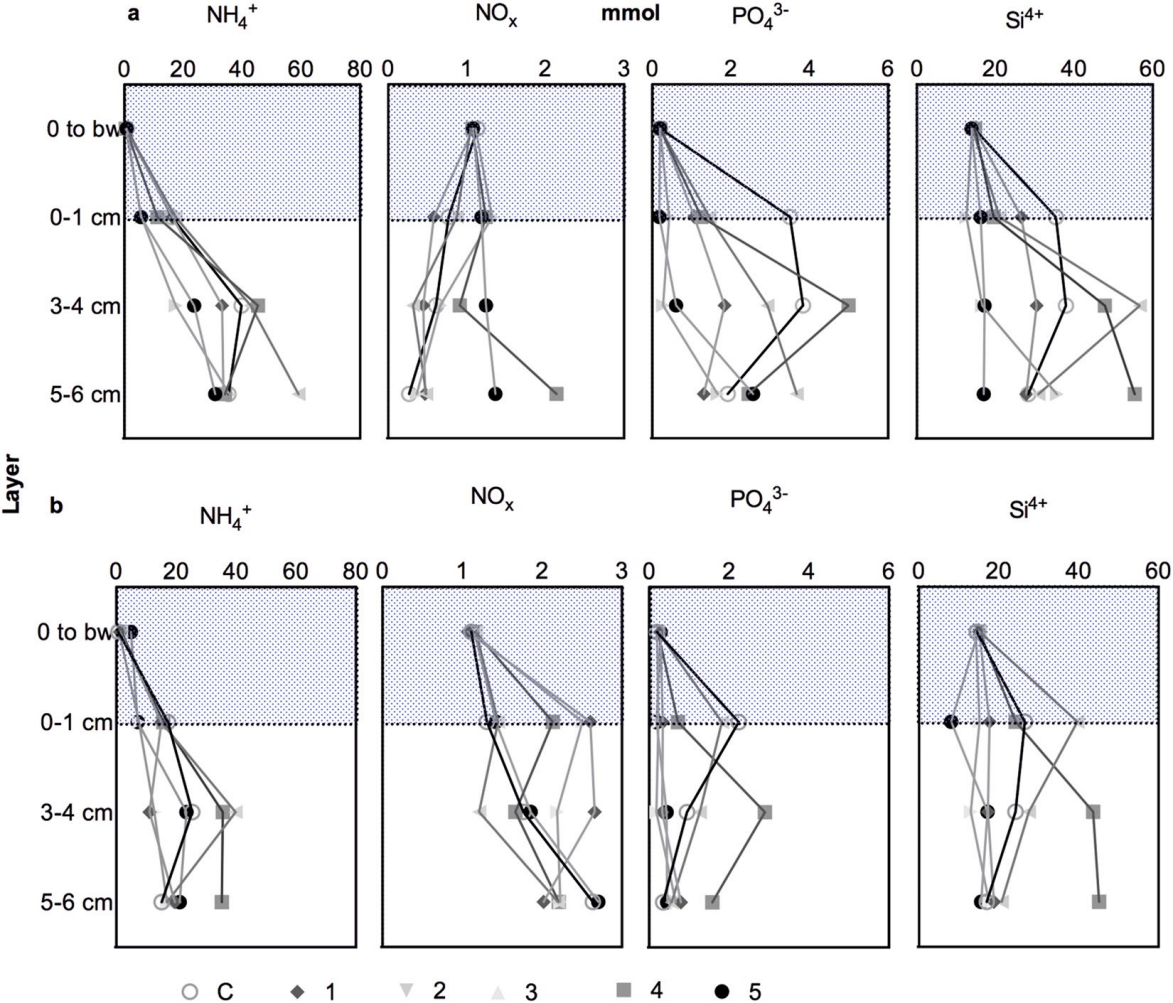
Pore water profiles of the different solutes at the start (**a**) and end (**b**) of the dark incubation of the sediment cores Ca, 1a, 2a, 3a, 4a and 5a. The blue color marks the water column. C = no worms added, 1 = 3 worms, 2 = 6 worms, 3 = 12 worms, 4 = 24 worms and 5 = 48 worms added.

At the start of the incubation, the pore water profiles of the control core Ca (Fig. 2a,b) showed profiles with increasing concentrations of ammonium, phosphate and silicate, and decreasing concentration of nitrate+nitrite from the surface down. During the incubation, the concentration of nitrate+nitrite (NO_x_) increased in all layers of the sediment. The concentrations of ammonium, phosphate and silicate, in contrast, decreased during the incubation in all layers of the sediment. Similar trends were apparent in all treatments, except for cores 3a and 5a. The concentration of NO_x_ decreased in the bottom water during the incubation in all treatments except for cores Ca and 1a, whereas the concentration of phosphate in the bottom water increased in all treatments except for cores Ca and 3a. Ammonium in the bottom water decreased in treatments 1a to 5a, and increased in core Ca, whereas the opposite was true for silicate.

Apart from the manipulated densities of *Marenzelleria* spp., the background communities present were highly variable across cores (see above) with high densities and large organisms potentially affecting pore water concentrations. The pore water control core (Ca), had a high biomass of *M. balthica* (155.6 g/m^2^) and a relatively high density of Hydrobiidae (2527 ind/m^2^) but very little other fauna. Core 3a had the third highest density of *Marenzelleria* spp. (2347 ind/m^2^) and a density of 1805 ind/m^2^ hydrobids, but otherwise very little other fauna, whereas core 5a had a large *Mya arenaria* weighing 6.0 g (1087.7 g/m^2^), the highest density of *Marenzelleria* spp. (9567 ind/m^2^), and the second-highest biomass of *M. balthica* (184.1 g/m^2^).

### Effects of macrofauna on bioturbation and solute fluxes

Detailed results including specified percentages, AIC and P-values and direction of the multiple partial correlations with the dbRDA-axes for individual predictors are presented in Table 2. Effects of macrofauna on bioturbation metrics, and of macrofauna and bioturbation metrics on solute fluxes are considered direct effects, effects of macrofauna through bioturbation on solute fluxes are considered indirect effects.

**Table 2:** DistLM results for all macrofauna densities and biomasses and bioturbation parameters as predictors of the solutes, and all macrofauna as predictors of the different bioturbation measures.

Available predictors	Response	Selection procedure	Selected predictors	AIC	Pseudo-F	P	Prop.	Cum-R^2^	Correlation with dbRDA-axis
Macrofauna + bioturbation	NO_x_	Sequential (forward)	Bioirrigation	194.60	3.88	0.06	0.17	0.17	−
			*Hediste* biomass	193.23	3.13	0.10	0.12	0.29	+
Macrofauna + bioturbation	NH_4_^+^	Sequential (forward)	*M. arctia* density	245.07	20.95	0.00	0.52	0.52	−
Macrofauna + bioturbation	PO_4_^3−^	Sequential (forward)	*Cerastoderma* biomass	−11.03	8.07	0.01	0.30	0.30	+
			D_b_^N^	−11.76	2.50	0.13	0.09	0.38	−
			*M. neg+vir* density	−13.53	3.34	0.09	0.10	0.48	+
Macrofauna+bioturbation	Si^4+^	Sequential (forward)	*Cerastoderma* biomass	297.19	12.55	0.00	0.40	0.40	−
			*M. neg+vir* density	296.44	2.51	0.13	0.07	0.47	−
			MPD	296.22	1.90	0.18	0.05	0.52	−
Macrofauna	MPD	Sequential (forward)	*Hediste* biomass	−8.12	10.12	0.01	0.35	0.35	+
			*Macoma* biomass	−10.67	4.35	0.06	0.13	0.47	+
			*Marenzelleria* biomass	−12.46	3.36	0.08	0.09	0.56	+
Macrofauna	SR	Sequential (forward)	*Marenzelleria* biomass	−106.09	12.14	0.002	0.39	0.39	+
Macrofauna	D_b_^N^	Sequential (forward)	*Marenzelleria* biomass	−20.68	2.92	0.11	0.13	0.13	+
			*Hediste* biomass	−21.50	2.59	0.12	0.11	0.24	+
			*Macoma* biomass	−22.13	2.27	0.15	0.09	0.33	+
Macrofauna	Bioirrigation	Sequential (forward)	*Marenzelleria* biomass	144.07	14.57	0.00	0.43	0.43	+
			*M. arctia* density	141.86	4.00	0.06	0.10	0.54	+
			*Cerastoderma* biomass	140.97	2.51	0.13	0.06	0.60	+

D_b_^N^ = biodiffusion coefficient, SR = % of surface reworked, MPD = maximum penetration depth, BI = bioirrigation. Macrofauna includes *M. arctia, M. neglecta* and *M. viridis* density, *Marenzelleria* spp. biomass, *M. balthica* biomass, *C. glaucum* biomass and *H. diversicolor* biomass.

### Direct effects of macrofauna on bioturbation metrics

**Figure 3:**
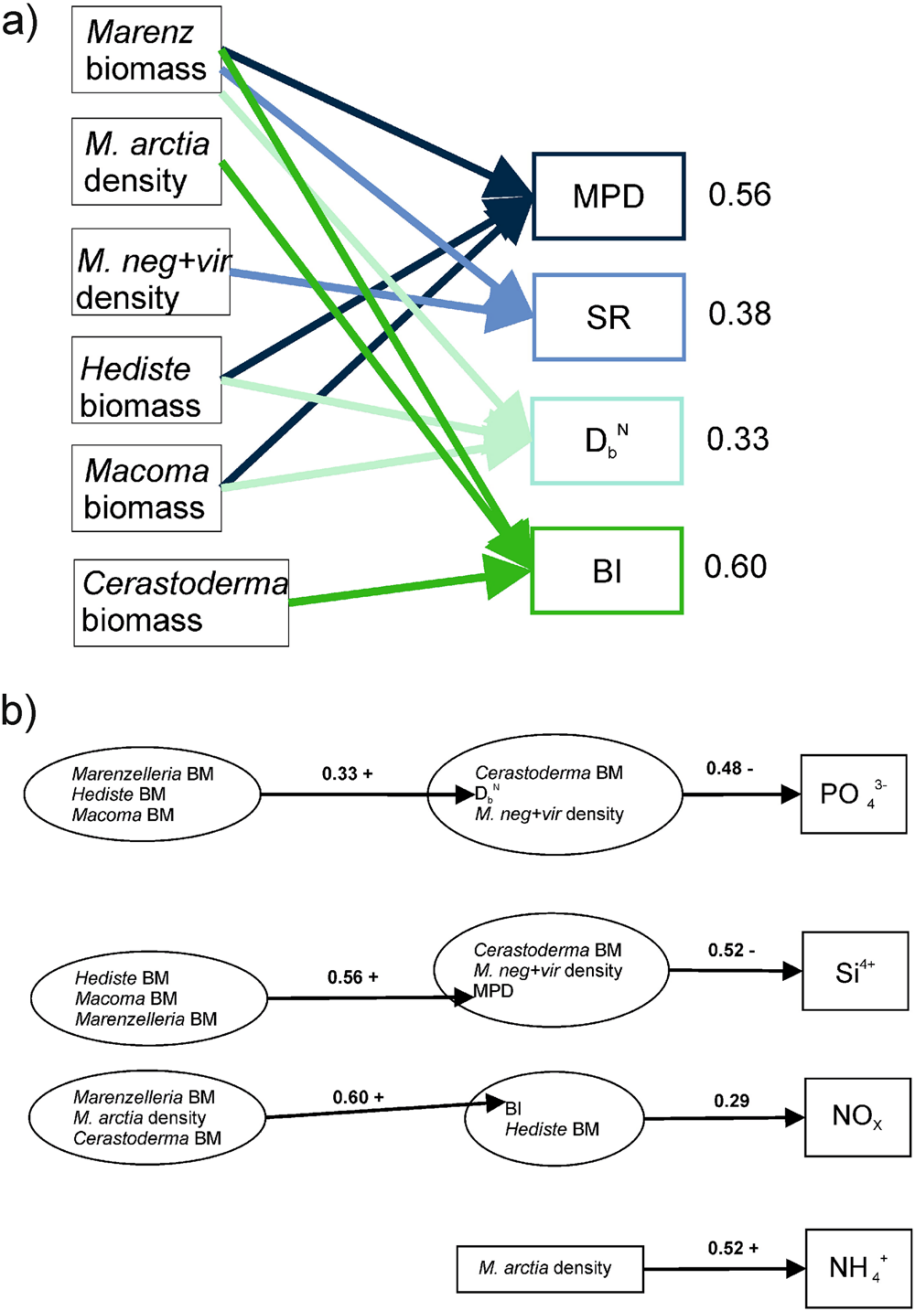
Direct effects of macrofauna on the different bioturbation metrics (**a**), and indirect effects of macrofauna through bioturbation on solute fluxes, and direct effects of macrofauna and bioturbation on solute fluxes (**b**) according to the DistLM models. The numbers indicate the amount of variation accounted for by the set of predictors, the sign indicates the direction of the correlation with the dbRDA -axis.

Macrofauna predicted in total 56% of MPD, 38% of SR, 33% of D_b_^N^, and 60% of bioirrigation (Fig. 3a). The significant predictors accounting for variation in MPD were the biomass of *H. diversicolor*, the biomass of *M. balthica* and the biomass of *Marenzelleria* spp., in SR *Marenzelleria* spp. biomass, and in bioirrigation *Marenzelleria* spp. biomass and the density of *M. arctia*. D_b_^N^ was not significantly predicted by any of the macrofauna at the P ≤ 0.10 -level. The combination of all macrofauna had a positive effect on all the bioturbation metrics (Fig. 3a).

### Direct effects of macrofauna and bioturbation on solute fluxes

**Figure 4:**
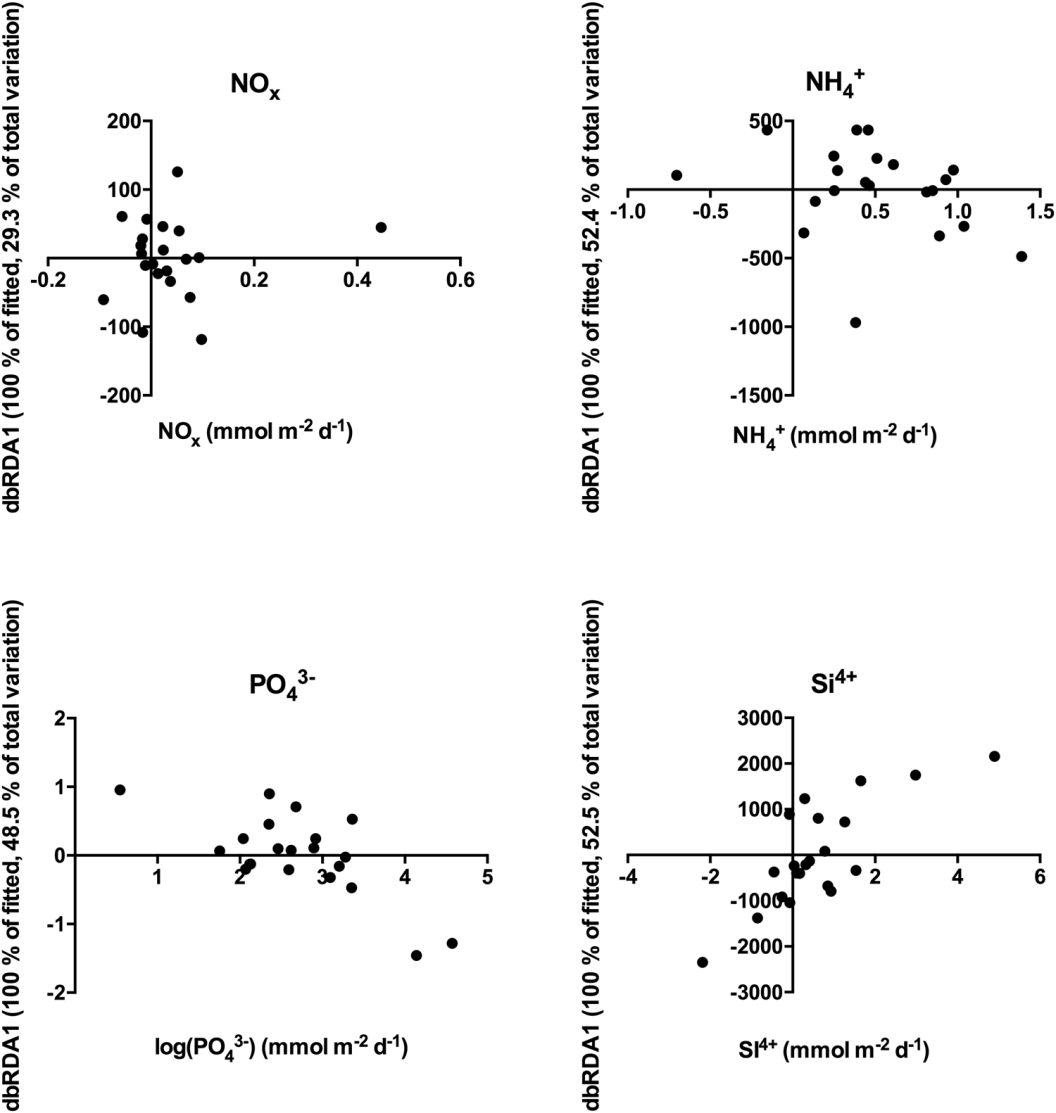
Results from the dbRDA-analysis with macrofauna and bioturbation metrics as predictors of the solute fluxes (see Table 2 for the predictors selected and the signs of the correlations with the dbRDA-axes). The y-axis represents the Euclidean distances between the sampled cores for the selected model.

The combination of all macrofauna and bioturbation metrics predicted 29% of the NO_x_ -, 52% of the NH_4_^+^ -, 61% of the PO_4_^3−^-, and 62% of the Si^4+^ -fluxes (Figs 3b, 4). The significant predictors selected accounting for the variation in NO_x_ -fluxes were bioirrigation and *H. diversicolor* biomass, for NH_4_^+^ -fluxes the density of *M. arctia*, for PO_4_^3−^-fluxes *C. glaucum* biomass and the density of *M. neg*+*vir* and in Si^4+^*-*fluxes *C. glaucum* biomass (Table 2). Bioirrigation and *H. diversicolor* biomass had opposite effects on NO_x_ -fluxes with bioirrigation decreasing and *H. diversicolor* increasing the fluxes. *M. arctia* increased the fluxes of ammonium. The biomass of *C. glaucum* and the density of *M. neg*+*vir* decreased the fluxes of phosphate, and the biomass of *C. glaucum*, density of *M. neg*+*vir*, and MPD increased the silicate fluxes (Figs 3b, 4).

## Discussion

Animal densities, biomasses and functional characteristics as well as their interactions affect sediment processes such as bioturbation and nutrient cycling^7,17^, and hence any changes in these parameters will influence ecosystem functioning. Changes in the abiotic and biotic environment can change the species-specific expression of functional traits^42,43^. Increasing biomass of the invasive polychaete *Marenzelleria* spp. significantly enhanced all bioturbation metrics examined, whereas the native fauna only had an effect on one or two individual bioturbation metrics. *Marenzelleria* spp. clearly dominated the bioirrigation pattern by predicting 43 out of 60% of total variation, which seemed to have, in the end, a negligible effect on all other but NO_x_ fluxes. Direct density-dependent effects of the taxon on the solute fluxes were demonstrable for NH_4_^+^ and PO_4_^3−^ and Si^4+^ fluxes. This has implications for ecosystem functioning especially in deeper, hypoxia-affected areas, where *Marenzelleria* spp. is sometimes the only macrofaunal taxon present. Particle reworking by all three species has previously been found to be negligible^35^. Our results, however, show that in the macrofauna community, the biomass of *Marenzelleria* spp. was the only significant predictor of the surface reworked and their biomass was also the predictor accounting for most of the variation in the biodiffusion coefficient, but these predictors were not important in predicting the variation in the solute fluxes. Macrofauna in general enhanced all bioturbation metrics, but individual species affected different bioturbation metrics differently, which in turn affected different solutes. The contrasting effects were associated with the variable importance of the separate bioturbation metrics on the individual solutes. The three different species of *Marenzelleria* had complex effects on the solute fluxes through the contrasting indirect effects mediated by their bioturbation. Future experiments with the three species separated from the beginning would be needed, but since their identification requires genetic analysis, conducting these kinds of experiments in areas with all three species co-occurring is challenging.

Of the individual solute fluxes in this study, phosphate and silicate responded to the same faunal components in opposite ways: the biomass of the cockle *C. glaucum* and the density of *M. neglecta* and *M. viridis* had a direct effect on the fluxes, which in the case of phosphate was a decrease in the efflux, but in the case of silicate an increase in the efflux. Increasing densities of cockles, the most significant predictor for both silicate and phosphate fluxes in this study, tend to increase sediment resuspension and erosion^44^, which may increase e.g. silicate fluxes^45^. Previous laboratory studies have found a positive effect of the bioirrigation of all the three *Marenzelleria* spp. species on phosphate effluxes, and an even greater enhancement by *M. viridis* and *M. neglecta* compared to *M. arctia*^36^, which partly contradicts our findings. The combination of *M. viridis* and *M. neglecta* explained more variation than *M. arctia* in phosphate fluxes, hence supporting the findings of Renz and Forster^36^, but on the other hand bioirrigation was not selected as a significant predictor of phosphate fluxes. However, an increase in the biodiffusion coefficient enhances phosphate efflux, and the biomass of *Marenzelleria* spp. enhances biodiffusion predicting the highest proportion, 13%, of the variation of all macrofauna, hence there might be an enhancement through density-dependent indirect effects on bioturbation. The positive correlation of *C. glaucum* biomass with the dbRDA -axis indicates that their increase decreases the efflux of phosphate. Highest phosphate fluxes tended also to occur when *C. glaucum* was absent, and *H. diversicolor* and *M. balthica* biomasses were high, highlighting the opposite roles of the large bivalves *C. glaucum* and *M. balthica* in this system. Large bivalves are known to have important effects on ecosystem functioning^46,47^. The importance of *C. glaucum* living at the surface sediment could be attributed to its role as a link between the benthic and pelagic environments^17^. Affecting only the top 2 cm of the sediment^3^, *C. glaucum* could enhance the buffering capacity of the top-sediment for phosphate by increasing the oxygenation of iron to Fe^3+^ that PO_4_^3−^ can adsorb to^48,49^, resulting in a decrease in the phosphate efflux. The decrease in phosphate efflux due to increasing densities of *C. glaucum* and *M. neg*+*vir* might also have been caused by their stimulation of bacterial growth leading to binding of the nutrients in the microbial biomass^19^.

Depending e.g. on their bioturbation mode and position in the sediment, different taxa affect the hydrodynamic conditions in the sediment and at the sediment-water -interface differently^9,46,50^. The functional roles of individual species might change when studied in the presence of other fauna^42^, which could thus change the direction and size of the effect of individual species: porosity of the sediment and the volume of oxygenized sediment can also increase due to activities of bivalves present in the experimental cores^17,51^, possibly modifying the effect of *Marenzelleria* spp. on solute fluxes in the already bioturbated sediment. Joensuu *et al*.^52^ found that in muddy sediments the densities of *Marenzelleria* spp. correlated negatively with the sediment erosion threshold, and positively with the erosion rate. Grazers, like *Peringia ulvae*, also numerous in our cores, can also cause disturbance and loosening of the sediment surface^53^. Increased resuspension can also lead to increased feeding by the suspension-feeding bivalves^50^, modifying organic matter availability and activity of microbial communities even further. The large role of *C. glaucum* in predicting the phosphate and silicate fluxes, 30 and 40% of variation accounted for, respectively, was nevertheless somewhat surprising given that in some previous experiments they have not been found to greatly affect solute fluxes or the microbial communities as the water pumped through its siphons is not in direct contact with the sediment biogeochemical environment^17^. The increase in surface reworking with increasing densities of *Marenzelleria* spp. suggests they could indirectly be able to modify the behavior of other species, such as *C. glaucum* or surface deposit feeders. The introduction of *Marenzelleria* spp. could thus have enhanced the strength of benthic-pelagic coupling in these environments through its effects on the native fauna. This is essential for the efficient functioning of the entire ecosystem^6^, and could have implications especially in the undisturbed areas, where the richness of native species is still high.

The relatively low level of variance explained, 29%, for nitrate/nitrite -fluxes by macrofauna and bioturbation compared to the other fluxes, 48 to 52%, indicates that other factors not used as predictors here, such as the microbial community composition^54^, abundance of meiofauna^55^ and availability of ammonium and oxygen^56^, are important for the cycling of these nutrients. Although not quantified here, meiofauna bioturbation is also known to stimulate bacterial denitrification in the Baltic Sea sediments, when large bivalves are absent^55^. Recent observational evidence, however, suggests that the influence of *Marenzelleria* spp. can be variable seasonally, with a potential for a very high importance in predicting NO_x_ fluxes at both shallow and deep sites associated with peaking densities, rising temperatures and organic matter input during the spring bloom^10^. The most important predictor for NO_x_ fluxes, bioirrigation, allows the overlying, oxygenized water to enter the anaerobic sediment zones, where solute exchange processes such as denitrification and nitrification take place, hence its importance in predicting the fluxes. *H. diversicolor* is a gallery-diffuser burrowing deep into the sediment, and is thus capable of irrigating large volumes of sediment. The presence of infauna in general stimulates mineralization, nitrification and denitrification processes and solute transport^57^. The different burrow ventilation and irrigation behaviors of macrofauna lead to differences in the microbial community affecting e.g. nitrification, which is e.g. enhanced in the presence of *H. diversicolor* but not of *M. viridis*^9^. The density of *M. arctia* was also the only significant predictor for ammonium fluxes predicting 52% of the variation, thus it could be the enhancement of the ammonium fluxes by increasing densities of this species that also has an indirect effect on the nitrate/nitrite fluxes. *Marenzelleria* spp. biomass and the density of *M. arctia* were also the most important predictors of bioirrigation, suggesting a significant indirect effect on nitrate/nitrite -fluxes through significantly enhancing bioirrigation. In previous experiments, *M. arctia* has been found to enhance ammonium efflux^58^ similarly to our results.

To understand the complex interactions of invasive species with their environment and ambient communities, experiments incorporating natural variability in a range of factors are imperative. This does, however, come with a cost as high variability can make interpretation difficult. In order to meet the assumptions of our statistical models, we often have to remove the most distinctive outliers, and prefer experimental setups with the least number of uncontrolled factors. By doing this we, however, reduce our ability to extrapolate to natural conditions. Even though the average might give us the standard effect, the endpoints of the continuum often contain very interesting information^59^. Due to the uncontrolled community composition, we were forced to remove four replicates from the overall analyses. This points out the difficulty of dealing with many (uncontrolled) sources of variation in these kinds of experiments. Accordingly, future experiments aiming at investigating the density-dependent effects of *Marenzelleria* spp. within the natural community should involve more replicates.

Colonization of previously hypoxic areas by *Marenzelleria* spp. could first lead to increased oxygenation and phosphorus binding^60^ and facilitate the return of the native fauna thus having a major role in the beginning. Later, when the sediment is reoxygenized, the effect of *Marenzelleria* spp. on phosphorus binding is decreased, and their effect on nutrient cycling will be dependent on environmental factors (e.g. organic matter input, temperature^10^), and macrofaunal community composition. The combined biomass of all three *Marenzelleria* species affects all the bioturbation metrics, whereas the other dominant members of the community only affect one or a few bioturbation metrics significantly. As a genus they could to some degree compensate for the loss of other members in the community, given their ability to enhance all bioturbation metrics. The three different species, on the other hand, prefer different habitats and differ in their population dynamics and in occupancy of sites spatially and temporally^33^, and differ in their effects on the individual bioturbation metrics, indicating that also the effect of the polychaetes on ecosystem functioning will vary accordingly. The uneven distribution of the different *Marenzelleria* -species in the cores could have affected the results, however, the proportions of the species densities in general represent those found in nature^33^, with highest adult densities expressed by *M. arctia*, and thus the resulting picture is also realistic. We also believe that because changes in *Marenzelleria* densities did not predict all of the variation in all the measured response variables, the range of variation in *Marenzelleria* density was not too high compared to other species present in the cores. The differential direct effects of the three species on the bioturbation metrics, and on the solute fluxes, differ: *M. arctia* seems to have a large effect on nitrogen cycling, whereas *M. neglecta* and *M. viridis* have an effect on phosphorus and silica cycles. Only a few sites will be occupied by all three species at the same time, thus the genus as a whole will only have a large effect on bioturbation at these few sites. Given the spatial separation of the populations in nature, there will also be a spatial separation in their effects on ecosystem functioning, with contrasting effects on different solutes, further depending on the structure of the surrounding community.

According to our results, the species and functional composition of the community affects the expression of the different bioturbation metrics present in the community, leading to changes in the direction and magnitude of the biogeochemical fluxes. Due to the different solutes being affected by different sets of macrofauna and bioturbation metrics, it is important to consider the whole community and not only taxa in isolation in biodiversity-ecosystem functioning experiments, as also interactions between the species and their functions could be significant for the outcome of the sedimentary processes. The magnitude and direction of the effect of *Marenzelleria* spp. on solute fluxes also seems to be dependent on the level of disturbance in the system, as increasing densities of the invader could e.g. enhance phosphate binding under hypoxic but not normoxic conditions. This suggests that in disturbed areas *Marenzelleria* spp. could act as a driver of change, whereas in undisturbed areas, and when disturbed areas possibly are recolonized by the native fauna and recover, its effect on e.g. biogeochemical cycling shifts towards being less important, and more affected by the native community composition. Thus, in the (deeper) seasonally hypoxic areas the consequences of the invasion of *Marenzelleria* spp. for ecosystem functioning can be positive, whereas in areas not affected by hypoxia they may not have added any significant value to the functional capacity of the community^25^ although seasonally their contribution to nutrient cycling also in these areas can be substantial^10^. Including environmental context, observations of natural history characteristics, behavior and interactions thus provides a way forward regarding our understanding of the complex ecosystem effects of invasive species.

## Material and Methods

The experiment was conducted in a temperature-controlled climate room at Tvärminne Zoological Station, University of Helsinki, in October 2014. The sediment cores used as experimental units and the *Marenzelleria* spp. added to the cores were collected from sites in close proximity to the station. All three species of *Marenzelleria* spp. occur at the sites, and the species identities were verified with genetic analyses (Bastrop *et al*. unpublished data).

### Sediment and worm collection

In total 24 sediment cores (plexiglass cores, 8.4 cm internal diameter) were obtained from a shallow, muddy site at five meters depth by SCUBA. An additional three sediment cores were obtained for sediment grain size, C/N and organic matter analyses. In the laboratory the cores were placed in a tank containing seawater with a flow-through in the tank and separately in each core. The cores were left to settle for one day before adding the worms.

The worms were collected at three different sites close to and at the sediment collection site using a Van Veen - grab at 5 to 10 m depth. The sediment was gently sieved and the intact worms collected from the sieve. The worms were then stored in jars with flow-through containing sediment until they were added to the experimental cores the following day.

### Experimental design

Each of the four replicate experimental blocks consisted of one control core with no added worms, and five density treatments with 3, 6, 12, 24 and 48 added worms per treatment corresponding to densities observed in nature and used in the study of Norkko and Reed *et al*.^38^. Hereafter the treatments will be referred to with C for control and numbers 1–5 for low to high density treatments, and letters a, b, c and d will denote the replicates. Worms were added into the cores and let to acclimatize and establish their burrows for 5 days. After worm addition the cores were fitted with nets to prevent escaping of the worms before they had burrowed. After addition of particle tracers at the sediment surface (see below), the experiment was let to run for nine days under flow-through incubation in an immersion tank supplied with natural running sea water, with a light-dark cycle corresponding to the ambient light cycle during that period (11 h/13 h light/dark regime). The temperature corresponded to the ambient temperature of the seawater during the experimental period (approximately 14 °C). Temperature, salinity and oxygen concentration of the incoming water and in the cores were followed daily. After nine days, the cores were fitted with lids provided with manual stirrers and incubated in darkness for four hours, and stirred manually with regular intervals to prevent gradients from forming in the cores. At start and end of the incubation samples of oxygen, NO_x_, NH_4_^+^, PO_3_^4−^ andSi^4+^ were obtained from each core. One core per treatment was also fitted with holes on the side for pore water extraction at 0-1, 1-2, 3-4 and 5-6 cm depth in the sediment. Pore water was extracted both at the start and at the end of the incubation and analyzed for NO_x_, NH_4_^+^, PO_3_^4−^ and Si^4+^. After the experiment the cores were sliced at 0.5, 1, 1.5, 2, 3, 4, 5, 6, 7, 8, 9, 11, 13, 15 cm, subsamples were obtained for particle mixing analyses (see below), the visible fauna was collected from the slices, and the rest of the core was sieved through a 0.5 mm sieve. The worms were stored separately in 98% ethanol to allow genetic analyses, and the rest of the macrofauna samples was stored in 70% ethanol and stained with rose Bengal. The samples were sorted under microscope, macrofauna identified at the lowest taxonomic level possible, counted and weighed. *Marenzelleria* spp. specimens were counted and weighed and sent to the University of Rostock for genetic analyses.

### Sediment particle mixing and bioirrigation

Sediment particle mixing was assessed through incubation of sediment cores using luminophores as sediment particle tracers^61^. After the 5-day acclimatization following the worm addition, the flow through was stopped and 2 g DW (Dry Weight) of luminophores (eco-trace®, environmental tracing systems, density = 2.5 g cm^−3^) were suspended, homogenized in seawater and spread at the sediment surface using a Pasteur pipette. Two size fractions of luminophores were used (“mud” with particle diameter between 10 and 70 µm and “sand” between 125 and 250 µm) in a ratio of 1.5 g “mud” and 0.5 g “sand” mimicking the grain size at the sediment collection site. Luminophores were allowed to settle for 1 h before flow-through was restarted. The incubation lasted 9 days.

At the end of incubation, a photograph of the sediment surface from above was taken. From this, the percentage of surface reworked (SR) was obtained by subtracting the surface still occupied by luminophores from the total surface area using image analysis and dividing this by the total surface area (see below). Cores were subsequently sliced (see above), slices were homogenized and an approx. 30 g aliquot of sediment sampled for luminophore counting after ensuring that no macrofauna were trapped. Sediment aliquots were freeze-dried and 1 g of dry sediment photographed under UV light using a digital camera. Luminophore pixels were counted after a binarization step (based on the RGB level) for each image corresponding to a single slice using image analysis software^62^. The relative concentrations of luminophores in each slice were then used to compute corresponding vertical depth profiles. These profiles were used for: (1) the determination of the Maximum Penetration Depth of the tracers during the course of the experiment (MPD), and (2) the mathematical fitting of a Continuous Time Random Walk (CTRW) model^63^ used to derive a single normal biodiffusion coefficient (D_b_^N^ in cm^2^ yr^−1^) value reflecting particle mixing intensity by the resident macrofauna^63,64^.

Bioirrigation was quantified in each core through the measurement of the decrease of an inert bromide (Br-) solute tracer spread in the overlying water. A known volume of stock NaBr solution (1 M) was introduced after the incubation to an initial Br- concentration of ca. 10 mM in the overlying water. Incubation then lasted 24 h during which overlying water was stirred using gentle air bubbling. After the addition of NaBr solution 5 ml of overlying water samples were taken at 0 (15 min), 6 and 24 hours. Samples were kept at 4 °C until analysis. Concentration of Br- ions in the water samples was analyzed spectrophotometrically^65^ at Tvärminne Zoological Station (Shimadzu UV-2501 PC) and the relation between bromide concentration in the overlying water and incubation time assessed using simple linear regression^66,67^. Bioirrigation rates were then given as a pore water exchange rate Q (in ml.d^−1^) calculated after^66^.

### Analyses

#### Solute fluxes

Oxygen samples were analysed by Winkler titration. Water column and pore water solutes were analysed at Tvärminne Zoological Station (Thermo Scientific Aquakem 250). Quantification limits for solutes were NH_4_^+^ 0.0001, NO_x_ 0.00003, PO_4_^3−^ 0.00003 and Si^4+^ 0.0007 mmol l^−1^. Solute fluxes measured in the experimental cores were calculated from the difference in the concentration between start and end samples as mmol m^−2^ d^−1^.While the oxygen concentration inside the experimental cores was allowed to decrease during the dark incubation following respiratory activities of the benthic community, the oxygen concentration never dropped below 56% saturation, and is therefore unlikely to have affected neither faunal behaviour nor chemical processes.

#### Sediment analyses

Organic matter content of the sediment was determined as loss on ignition (LOI%). The samples were first dried at 60 °C for 48 h and thereafter combusted at 500 °C for 3 h. For grain size analysis, sediment was first placed in 6% hydrogen peroxide –solution to remove the organic matter. Thereafter the sediment was sieved through a series of sieves (2, 1, 0.5, 0.25, 0.125 and 0.063 mm), the different fractions were dried at 60 °C for 48 h or until dry, and the dry weights of the fractions were measured. The C/N -ratio of the sediment was analysed at Tvärminne Zoological Station with a Europa Scientific ANCA-MS 20-20 15 N/13C mass spectrometer after removal of carbonates from the sediment with HCl (aq).

#### Statistical analyses

We used a series of multivariate multiple regression analyses (DISTLM, PERMANOVA + for PRIMER^68^) with distance-based redundancy analysis, dbRDA, to distinguish the effect of *Marenzelleria* spp. and other macrofauna on different solute fluxes and bioturbation parameters. The parameter % of surface reworked was arcsine transformed. The series of models tested the direct effects of bioturbation metrics and macrofauna on solute fluxes and the direct effects of macrofauna on the different bioturbation metrics. Forward and backward selection procedures were used, and Akaike Selection Criterion (AIC) was used as the selection/stopping criterion. The macrofauna used as predictors were the biomass of *M. balthica, Cerastoderma glaucum, Hediste diversicolor* and *Marenzelleria* spp., and the densities of *M. arctia*, and *M. viridis*+*M. neglecta*+hybrids of these combined (*M. neg*+*vir* from hereon). Due to highly variable densities or highly correlated density and biomass that would have had a disproportionate effect in the multivariate analysis, we decided to use biomass for large clams and *Hediste* and the combination of all the *Marenzelleria* species. It was also not possible to separate the biomass of *Marenzelleria* on the species level. To explore species-specific density-dependent effects of *Marenzelleria* these were included in the analysis as separate predictors. Despite the high abundance of hydrobid snails (*Peringia ulvae* and *Ecrobia ventrosa*) in the cores, these did not significantly affect any of the bioturbation metrics or solutes, and were thus removed from the analyses. In the first series of models, the analysis was allowed to select predictors from macrofauna and bioturbation metrics best accounting for variation in the individual solute fluxes. In the second series of models, the analysis was allowed to select predictors from macrofauna best accounting for variation in the different bioturbation metrics. The effect of macrofauna alone excluding bioturbation metrics, and of bioturbation metrics alone excluding macrofauna on solute fluxes was also tested but since the results from these analyses corresponded to the analyses with macrofauna and bioturbation metrics combined, these will not be further discussed. In the analysis, the response variable is a matrix based on Euclidean distances between the core-wise samples of either the different solute fluxes or the bioturbation metrics. The predictor variables are automatically standardized in the analysis, howerer transformation was required for some variables to include non-linear responses. The biomasses of *C. glaucum* and *H. diversicolor* were log(x + 1) -transformed, whereas the densities of *Marenzelleria* spp. and the biomass of *M. balthica* were square root -transformed. The biomass of *Marenzelleria* spp. did not require transformation. The response variable phosphate was log(x + 10) transformed. The other solutes or the bioturbation metrics were not transformed. The relative impact of the predictors was assessed by examining the direction and magnitude of the correlation coefficients. Therefore we believe the significance level 0.1 is sufficient to regard the predictor variables as possibly having an effect on the response variables. The pore water profiles were analysed visually.

### Data availability

The datasets generated during and/or analyzed during the current study are available from the corresponding author on reasonable request.
